# Factors associated with postnatal care utilization among postpartum women in Ethiopia: a multi-level analysis of the 2016 Ethiopia demographic and health survey

**DOI:** 10.1186/s13690-020-00415-0

**Published:** 2020-04-15

**Authors:** Bezawit Adane, Girmatsion Fisseha, Getaw Walle, Melaku Yalew

**Affiliations:** 1 Department of Biostatistics and Epidemiology, School of Public Health, College of Medicine and Health Sciences, Wollo University, Dessie, Ethiopia; 2 Department of Reproductive and Family Health, School of Public Health, College of Medicine and Health Sciences, Mekelle University, Mekelle, Ethiopia; 3 Department of Reproductive and Family Health, School of Public Health, College of Medicine and Health Sciences, Wollo University, Dessie, Ethiopia

**Keywords:** Postnatal care utilization, Factor associated, Multi-level analysis, Ethiopia

## Abstract

**Background:**

Most postpartum women and newborns do not utilize postnatal care due to less emphasis given especially in developing countries. Understanding individual and community-level factors associated with postnatal care will help to design appropriate strategies and policies for improving service utilization. Therefore, this study aimed to assess individual and community-level factors associated with postnatal care utilization in Ethiopia.

**Method:**

This study used the Ethiopian Demographic and Health Survey (EDHS) data of 2016. A total of 4489 women who gave birth 2 years before the survey were included. Two-stage stratified cluster sampling technique was used. The analysis was done using Stata version 14.0 after checking for basic assumptions of multilevel logistic regression. Multilevel mixed-effects logistic regression was used to identify determinants of postnatal care utilization. An adjusted odds ratio with a 95% confidence interval was used to show the strength and direction of the association.

**Results:**

Husband with secondary education [AOR = 0.17, 95% CI = (0.04, 0.68)], four or more antenatal care visit [AOR = 10.77, 95% CI = (2.65, 43.70)], middle wealth quintile [AOR = 3.10, 95% CI = (1.12, 8.58)] were individual level factors. Community level education [AOR = 2.53, 95% CI = (1.06, 6.06)] and community level of health service utilization [AOR = 2.32, 95% CI = (1.14, 4.73)] were the predictors at community level.

**Conclusion:**

Wealth index, number of antenatal care visits, husband education, community level of education and health service utilization were significantly associated with PNC service utilization. Provision of quality antenatal care, improvement of the educational status of women and husband involvement in PNC are important strategies to increase PNC service utilization.

## Background

Postnatal care (PNC) is a constellation of critical care given after childbirth to mothers and her newborn [1]. The postpartum period starts from 1 h after the delivery of the placenta and ends 6 weeks after delivery [2]. According to World Health Organization (WHO), the mother and newborn should receive PNC during the first 24 h after birth if birth is in a health facility. Whereas, the first postnatal contact should be as early as possible within 24 h if the birth is at home and at least three additional contacts are recommended for all mothers and newborns on day 3 (48-72 h), between day 7 to day 14 and 6 weeks after birth [3].

It is the most important maternal health care service for the prevention and management of physical and mental impairment as well as a disability that occurs during the postnatal period [3]. It is also very essential for the health of both the mother and the child [4, 5]. Despite its importance, it is the most neglected maternal health service particularly in developing countries [6–9]. The Demographic and Health Survey (DHS) results of different African countries indicated that only 36% of women had a postnatal visit within 2 days [10] but, it was much less in Ethiopia which was 17% according to 2016 EDHS [11].

The postnatal period is a risk period in which most of the maternal and neonatal mortality happen, especially in the first 24 h [3]. WHO reported that 28% of maternal mortality occurred in the intra-partum and immediate postpartum period and the rest 36% were between 24 h to 42 days postpartum [12]. Out of all live births, an estimated 3.7 to 4 million deaths occur in the first 28 days of life [13]. About half of these deaths occur on the first day of life, and more than two-thirds within the first week of life [14].

Although, policies and programs have largely stressed this critical period still the implementation is poor [3]. Sustainable Development Goals (SDG) came into action particularly SDG-3 which provides a new strategy, by the year 2030 to reduce neonatal and maternal mortality to below 12 per 1000 and 70 per 100,000 live births respectively [15]. Besides, Ethiopian government adopts Health Sector Transformation Plan (HSTP) which aims to decrease maternal mortality ratio (MMR) from 420 to 199 per 100,000 live birth and infant and neonatal mortality rates from 44 and 28 to 20 and 10 per 1000 live births by 2020 [16, 17]. Regardless of efforts made by the Ethiopian government, the use of postnatal care services is still very low [11].

The factors which determine the utilization of PNC services differ from place to place, from individual to community which is affected by the culture and belief of women [18–24]. Even though the factors for PNC service utilization were from different levels, those previous studies conducted in Ethiopia simple regression model of analysis and the sample sizes were covering only specific or local areas [25–27]. Since the assumption of independence among observations within the same cluster and equal variance between clusters is violated in the case of nested data. Regressing factors from different levels by using a standard binary regression model leads to bias and loss of power. In addition to this, such type of analysis may result in either atomistic or ecological fallacy. So, this study aimed to assess individual and community-level factors associated with postnatal care utilization among postpartum women using the 2016 EDHS Dataset.

## Methods and materials

### Study area and design

A cross-sectional study design using secondary analysis of 2016 Ethiopia DHS data was used. The 2016 survey was the recent EDHS Which was conducted by the Central Statistical Agency (CSA), Federal Minister of Health (FMoH) and Ethiopian Public Health Institute (EPHI) with technical assistance from the International Classification of Functioning (ICF) [11]. Ethiopia is located in the North-Eastern part of Africa, also known as the horn of Africa, lies between 3^0^ and 15^0^ North latitude and 33^0^ and 48^0^ East longitudes. It borders six countries - Eritrea, Djibouti, Somalia, Kenya, South Sudan, and Sudan and it covers an area of 1.1 million square kilometers ranging from 4, 620 m above sea level to 148 m below sea level [28]. It has a total of 110,582,083 populations, of which 55,047,955 were women [29].

### Study population and sample size

**Fig. 1:**
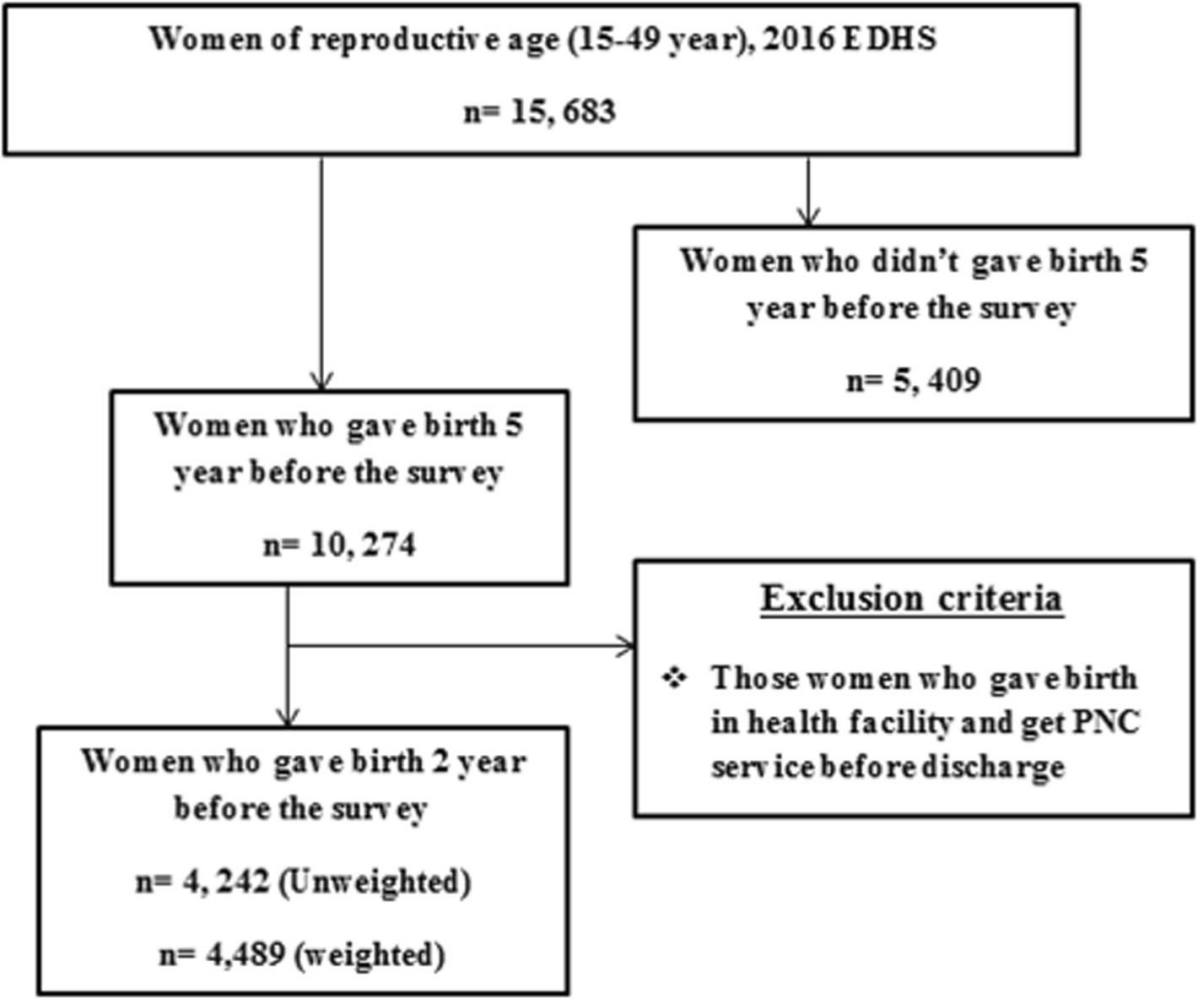
--+Sampling and exclusion procedures to identify the final sample size in 2016 EDHS

A total of 4489 women who gave birth in the 2 years before the survey (January 18, 2016) from the Ethiopian DHS 2016 dataset were included from nine geographical regions and two administrative cities of Ethiopia. Women who gave birth at the health facility and get PNC service before being discharged from the health facility were excluded from the analysis (Fig. 1).

### Variable measurement

The outcome variable (PNC utilization was measured if a woman received at least one visit/care during the first week after discharge or delivery at home [30]. Women’s autonomy was measured by the decision-making power in the household using the following three questions. Who decides matters about the woman’s health, major purchases, and visits to friends or family? A woman, who made more than one decision, either alone or jointly with her husband, was categorized as having high autonomy, a woman who made one or no decision making was categorized as having autonomy [31]. Attitude towards wife-beating was measured based on five hypothetical scenarios: did she goes out without telling him, Did she neglects the children, Did she argues with him, did she refuses to have sexual intercourse with him and did she doesn’t cook food properly. Based on the above listed scenarios, if the woman agreed that her husband had a right to beat her in any of these five hypothetical scenarios, she was classified as having favorable attitudes toward domestic violence against women. If she doesn’t agree with any of these hypothetical scenarios, she was classified as having opposing attitudes toward domestic violence [31].

### Data processing and analysis

Data cleaning was conducted to check for consistency and missing value. Recoding, labeling, and exploratory analysis were performed. Categorization and re-categorization were done for different variables according to the result of different kind of literature. Descriptive statistics were used to present frequencies, with percentages in tables, graphs and using texts. For analysis, Stata/SE version 14.0 was used. Sample weight was used to compensate for the unequal probability of selection between the strata that were geographically defined, as well as for non-responses. Any further explanation regarding the sample weighting procedure can be founded in the EDHS methodology report [11].

Multilevel analysis was conducted after checking that the data was eligible for multilevel analysis (Intra-class Correlation Coefficient (ICC) greater than 10% (ICC = 42%)). Since DHS data are hierarchical, i.e. individuals (level 1) were nested within communities (level 2), a two-level mixed-effects logistic regression model was fitted to estimate both independent (fixed) effects of the explanatory variables and community-level random effects on postnatal care utilization. Because the log of the probability of PNC utilization will be modeled using a two-level multilevel model as follows [32]:

$$ \mathrm{Log}\ \left[\frac{\varPi ij}{1-\varPi ij}\ \right]={\upbeta}_0+{\upbeta}_1\ {\mathrm{X}}_{\mathrm{ij}}+{\mathrm{B}}_2\ {\mathrm{Z}}_{\mathrm{ij}}+{\upmu}_{\mathrm{j}}+{\mathrm{e}}_{\mathrm{ij}} $$

Where, i and j are the level 1 (individual) and level 2 (community) units, respectively; X and Z refer to individual and community-level variables, respectively; πij is the probability of PNC utilization for the i^th^ women in the j^th^ community; the β’s will be the fixed coefficients. Whereas, β0 is the intercept-the effect on the probability of PNC use in the absence of influence of predictors; and uj showed the random effect (effect of the community on PNC) for the j^th^ community and eij showed random errors at the individual levels. By assuming each community had different intercept (β0) and fixed coefficient (β), the clustered data nature and the within and between community variations will be taken in to account.

**Fig. 2:**
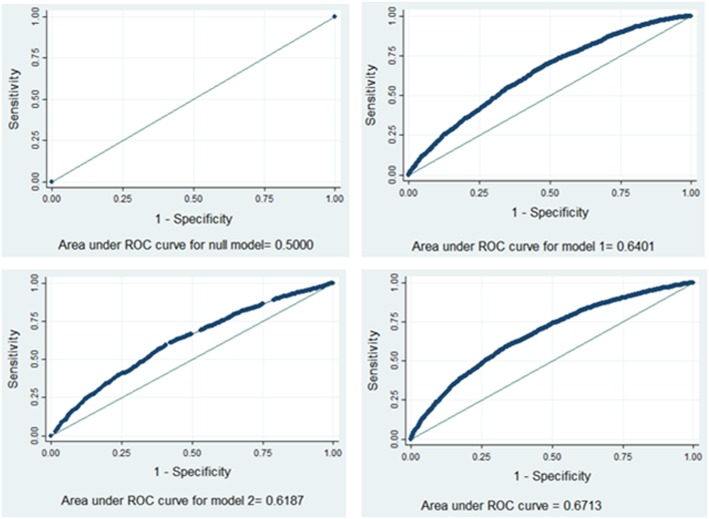
The area under the ROC curve for each model (null model, model 1, model 2 and mode 3) in 2016 EDHS

During analysis first, bivariable multilevel logistic regression was fitted and variables with *p*-value less than 0.2 were selected to build the 3 models (model1–3). Then the analysis was performed in four steps: Model 0 (empty model/ without explanatory variable); Model 1 (only individual-level factors) Model 2 (only community-level factors); and Model 3 (both individual and community-level factors). The measures of association (fixed-effects) estimate the associations between the likelihood of women to use PNC and various explanatory variables were expressed as Adjusted Odds Ratio (AOR) with their 95% confidence level. A variable in which the confidence interval does not include the null value and *p*-value < 0.05 was used to define statistical significance. The measures of variation (random-effects) were reported using ICC, Median Odds Ratio (MOR) and proportional change in variance (PCV) to measure the variation between clusters. The ICC was used to explain how much the observation in the same cluster resembled each other while MOR is a measure of unexplained cluster heterogeneity. The ICC was calculated as follows: [ICC= $\frac{\ {\delta}^2}{\ {\delta}^{2+\frac{\pi^2}{3}}}$], *δ*^2^ where is the estimated variance of clusters. MOR is defined as the median value of the odds ratio between the area at highest risk and the area at the lowest risk when randomly picking out two areas and it was calculated using the formula [MOR = exp.($\sqrt{2{x\delta}^2+0.6745}$) ≈ exp(0.95*δ*)]. In this study, MOR shows the extent to which the individual probability of using PNC is determined by the residential area. The proportional change in variance (PCV) measures the total variation attributed by individual-level factors and area-level factors in the multilevel model. The formula for PCV and MOR has been described elsewhere [32]. The presence of Multicollinearity was checked among independent variables using standard error at the cutoff point of _±_2 and there was no Multicollinearity. The log-likelihood test and area under the ROC (Receiver Operating Characteristic) curve were used to estimate the goodness of fit of the adjusted final model in comparison to the preceding models. The area under the curve of each subsequent model was computed (Fig. 2).

## Results

### Socio-demographic characteristics of the respondent

The total numbers of women who gave birth within 2 years before the survey and included for analysis were 4489 mothers. Out of this, 3297 (73.4%) were founded in the age group 20–34 years. Looking at the age at first marriage, 1769 (39.40%) married between ages 16–19 years. About 4269 (95%) were currently married and 1646 (36.6%) were employed. From the total husbands of the women 1924 (45.35%) had no education and 3761 (88.60%) husbands were employed, 1889 (42.08%) women had birth orders 2 to 4, about 1602 (37.61%) women gave birth at the health facility. Three thousand three hundred seventeen (73.90%) wanted their last child (Table 1).

**Table 1:** Socio-demographic characteristics of women in Ethiopia who gave birth within 2 years before the survey, EDHS 2016 (*n* = 4489)

Variables	Category	Frequency	Percentage
Age	15–19	287	6.39
	20–34	3297	73.46
	35–49	904	20.14
Marital status	Never in union	32	0.70
	Formerly married	188	4.18
	Currently married	4269	95.12
Age at first marriage	< 16 years	1708	38.05
	16–19 years	1769	39.40
	> 19 years	1013	22.55
Educational status	No education	2736	60.95
	Primary	1355	30.18
	Secondary	268	5.98
	Higher	130	2.89
Occupation	Not employed	2843	63.34
	Employed	1646	36.66
Husband Education	No education	1924	45.35
	Primary	1710	40.29
	Secondary	393	9.27
	Higher	216	5.09
Husband occupation	Not Employed	484	11.40
	Employed	3761	88.60

### Reproductive history

Regarding ANC visit, 1595 (35.54%) of women had no ANC visit, whereas 1495 (33.30%) had four and above ANC visits. About 4369 (97.34%) women gave birth via cesarean section, and 489 (11.47%) had low women autonomy. From the total last newborns, 2311 (51.48%) were female and about 1812 (40.82%) had average size at birth. Out of the total study participants 3953 (88.07%) resided in a rural area, 2710 (60.38%) responded that distance to the health facility was far and 2006 (44.69%) resided in Oromia region. The median and interquartile range (IQR) for the proportion of women employed and the proportion of women who had a negative attitude towards wife-beating were 28.57 (36.7) and 60 (41.6) respectively (Table 2).

**Table 2:** Obstetric and related characteristics of women in Ethiopia who gave birth within 2 years before the survey, EDHS 2016 (*n* = 4489)

Variables	Category	Frequency	Percentage
Birth order	1	912	20.31
	2–4	1889	42.08
	≥5	1688	37.61
Place of delivery	Home	2887	64.31
	Health facility	1602	35.69
The last child wanted	No	1172	26.10
	Yes	3317	73.90
No of ANC visit	0	1595	35.54
	1–3	1399	31.16
	≥4	1495	33.30
Mode of delivery	Cesarean section	4369	97.34
	Vaginal	119	2.66
Women autonomy	Low	489	11.47
	High	3779	88.53
Attitude towards wife-beating	Opposing	2485	55.51
	Favorable	1991	44.49

### Individual and community level factors for PNC utilization (fixed effects)

After adjusting for individual and community level factors (model 3), the educational status of husband, numbers of ANC visits, household wealth index and community level of education and health service utilization were significantly associated with PNC utilization. Independent of other factors, women whose husband had secondary education were 83% less likely to use PNC as compared to women whose husband had higher education [AOR = 0.17, 95% CI = (0.04, 0.68)]. Those women who had 1–3 ANC visits were 7 times more likely to utilize PNC service as compared to women who had no ANC visit [AOR = 7.27, 95% CI = (1.81, 29.27)]. likewise, women who had four and above ANC visits were 11 times more likely to use PNC service as compared to women without ANC visit [AOR = 10.77, 95% CI = (2.65, 43.70)]. Looking at the wealth of household, women who live in the middle wealth quintile were 3 times more likely to utilize PNC than women who live in the poorest wealth quintile [AOR = 3.10, 95% CI = (1.12, 8.57)]. Among community-level variables, community level of education and community level of health service utilization showed a statistically significant association. Those women who lived in a community with a high level of education were 2.5 times more likely to use PNC as compared to women who lived in a community with a low level of education [AOR = 2.53, 95% CI = (1.06, 6.06)]. Similarly, women who lived in the community who had a high level of health service utilization were 2 times more likely to have PNC utilization as compared to women who lived in community with a low level of health service utilization [AOR = 2.32, 95% CI = (1.14, 4.73)] (Table 3).

**Table 3:** Multilevel logistic regression analysis of individual and community-level factors associated with PNC in Ethiopia, EDHS 2016

Individual and community level characteristics	COR (95% CI)	Model 0 ***n*** = 4242 ICC = 42%	Model 1 AOR (95% CI) ***n*** = 4000	Model 2 AOR(95% CI) ***n*** = 4206	Model 3 AOR (95% CI) ***n*** = 3967
**Educational Status of women**					
No education	1		1		1
Primary	1.19 (0.63, 2.25)		0.98 (0.47, 2.07)		0.86 (0.40, 1.86)
Secondary	1.95 (0.76, 4.97)		1.15 (0.41, 3.23)		0.94 (0.35, 2.51)
Higher	3.97 (1.09,14.35)		2.92 (0.78,11.01)		1.98 (0.54,7.31)
**Occupation of women**					
Not employed	1		1		1
Employed	1.66 (0.91, 3.03)		1.43 (0.77, 2.66)		1.26 (.62, 2.55)
**Place of delivery**					
Home	1		1		1
Health facility	2.25 (1.18, 4.27)		1.19 (0.58,2.45)		1.01 (0.48, 2.12**)**
**Number of ANC visits**					
No	1		1		1
1–3	7.27 (2.29, 22.97)		8.3 (2.12, 32.35)		7.27 (1.8,29.27)*
> =4	12.0 (3.84,37.51)		12.24 (3.1, 8.24)		10.8 (2.65,43.7)*
**Women autonomy**					
Low	1		1		1
High	2.64 (.87, 7.96)		2.02 (0.58,6.7)		2.09 (0.65, 6.74)
**Media exposure**					
No exposure	1		1		1
Low exposure	2.42 (1.23, 4.76)		1.43 (0.70, 2.91)		1.27 (0.62,2.60)
High exposure	1.11 (.56, 2.20)		0.4 (0.19,1.18)		0.47 (0.18, 1.23)
**Wealth Index**					
Poorest	1		1		1
Poorer	1.19 (0.39,3.56)		0.97 (0.32, 2.9)		0.96 (0.32,2.84)
Middle	4.08 (1.58,10.52)		2.87 (1.08, 7.7)		3.10 (1.12,8.57)*
Richer	3.43 (1.25, 9.44)		2.18 (0.76, 6.3)		2.18 (0.69,6.81)
Richest	4.89 (1.77,13.54)		2.73 (0.8, 9.29)		1.99 (0.44,9.09)
**Husband education**					
No education	0.28 (0.11, 0.77)		0.97 (0.28, 3.30)		1.02 (0.29,3.61)
Primary	0.35 (0.13, 0 .92)		0.91 (.28, 2.93)		0.97 (0.28,3.26)
Secondary	0.09 (0.03, 0.34)		0.17 (0.04, 0.68)		0.17 (0.04,0.68)*
Higher	1		1		1
**Place of residence**					
Urban	1			1	1
Rural	0.34 (0.19,0 .58)			0.91 (0.40,2.05)	0.82 (0.25, 2.64)
**Distance to the HF**					
Far	1			1	1
Near	2.57 (1.39, 4.74)			2.03 (1.05,3.92)	1.92 (0.97, 3.78)
**Proportion of women employed**©	1.02 (1.01,1.03)			1.01 (1.00,1.02)	1.01 0(.99, 1.02)
**Community-level of poverty**					
Low	1			1	1
Middle	0.62 (0.31, 1.17)			1.32 (0.60,2.82)	1.54 (0.68, 3.44)
High	0.31 (0.16,0.60)			0.92 (0.38,2.16)	1.59 (0.60, 4.23)
**Community-level of education**					
Low	1			1	1
Middle	1.64 (0.68,3.88)			1.32 (0.58,3.01)	1.02 (0.44, 2.34)
High	4.29 (2.05, 8.97)			2.56 (1.14,5.73)	2.53 (1.06, 6.06)*
**The proportion of women with the opposite attitude to wife-beating**©	1.01 (1.00,1.02)			1.0 (0.99,1.01)	1.00 (0.98, 1.01)
**Community-level of HSU**					
Low	1				1
High	3.75 (1.93,7.26)			2.25 (1.16, 4.4**)**	2.32 (1.14, 4.73)*

Key: * = *P*-value

< 0.05, ** = *P*-value < 0.01, AOR = Adjusted odds ratio and© = Continuous Variable, HSU = Health service utilization 1 = Reference

### Random effects (measures of variation)

There was a significant variation in the utilization of PNC across the communities (clusters). The ICC showed that 42% of the variation in utilization of PNC was linked to community-level factors. The full model, after adjusting for individual and community level factors, showed that the variation in PNC utilization across communities remained statistically significant. About 37% of the PNC use variation across communities was explained in the full model. Besides, the MOR confirmed that PNC utilization was attributed to community-level factors. The MOR for PNC utilization was 3.97 in the empty model; these show that there was variation between communities (clustering) (Table 4).

**Table 4:** Result from a random intercept model (a measure of variation) for PNC at cluster level by multilevel logistic regression analysis, EDHS 2016

Measure of variation	Model 0 (Null model) (95% CI)	Model 1 (95% CI)	Model 2 (95% CI)	Model3 (Full model) (95%CI)
Variance	2.37	1.77	1.52	1.50
Explained variation (PCV) (%)	Ref.	25	35	37
ICC (%)	42 (30.00, 53.99)	35 (24.62, 45.37)	32 (22.33, 41.66)	31 (21.39, 40.61)
MOR	3.97 (3.85, 4.09)	3.42 (3.31, 3.2)	3.18 (3.08, 3.27)	3.15 (3.05, 3.24)
Model fitness				
Log-likelihood	− 552.68	− 466.41	− 525.29	− 451.52

Model 0 = without independent variables (null model), Model 1 = only individual-level variables, Model 2 = only community-level variables, Model 3 = both individual and community-level variables, *PCV* Proportional change in variance, *ICC* Intra-class correlation coefficient and *MOR* Median odds ratio

## Discussion

In this study, the educational status of the husband, number of ANC visits, wealth index, community level of education and community level of health service utilization were significant determinants of PNC utilization. This study showed that the ANC visit was positively associated with PNC utilization. The finding is similar to different studies conducted in different parts of Ethiopia [33, 34]. It is also consistent with a study conducted in Tanzania [35]. There was also a similar report in studies Nepal [36] and India [6]. The possible reason behind this could be, the characteristics that predispose women to seek ANC service could also make them more likely to seek PNC service. Besides, those women who were using ANC service could be familiar with other maternal health service and it might also create an opportunity for health professionals to provide counseling regarding the significance of PNC services [23, 33].

The household wealth index was another positively associated variable. Postpartum women who belong to the middle household wealth quintile were more likely to use PNC. The finding is similar to different studies conducted in Gondar [37], Swaziland [38], and Nepal [36]. The possible explanation for this might be women would be improved educationally, economically and socially which would create a better opportunity for seeking health care [31]. Moreover, it would provide women with economic opportunities to afford the cost of health care for health services that may not be free in public health institutions [24]. On the other hand, the poorest people would be reluctant about their health care because they might be hopeless since they are still running for their basic needs. But a study conducted in Nigeria showed that women who belonged to the poorest wealth quintile were more likely to use PNC [39]. The discrepancy might be due to the difference in socio-demographic characteristics, method of analysis, sample size and study time variation.

Husband education was also positively associated with PNC utilization. This result is consistent with studies conducted in Amhara regional hospitals [27], a systematic review in developing countries [8] and West African countries [24]. There was also a similar report from studies conducted in Nigeria [39] and Nepal [40]. The possible justification behind this could be educated husband may have better communication with their wives and they may have the willingness to discuss the use of PNC service as well as other maternal health services. This may also provide more autonomy to their wives [33].

Community-level of education was one of the community-level variables which had a significant association with PNC. Women who live in a community with high education levels were more likely to have PNC utilization. This finding is similar to studies conducted in West African counties [24] and India [41]. The reason for this could be the women who live in such a community would be better informed about the benefits of PNC utilization as well as health risks of non-utilization of the service and more empowered to seek healthcare [24, 31]. Furthermore, it could be because of education has a valuable input in enhancing female autonomy that helps women develop greater confidence and capability to make decisions about their health [26]. Community-level of health service utilization was also significantly associated with PNC. Likewise, a study conducted in Tanzania revealed similar findings [42]. This could be because of the community which has a good habit of health service utilization could have better behavior to utilize PNC services and health providers might recommend them about PNC while receiving other health services.

The study also indicated that there was a statistical significant correlation between observations found in the same cluster. About more than one-third of PNC use variation across communities was explained in the full model. The MOR for PNC utilization showed that there was variation between communities (clustering).

This study was conducted by using multilevel mixed-effects logistic regression analysis that can be able to identify the multilevel factors of PNC service utilization and provides important insight to design interventions. Not only this, but it is also more representative of the entire population of Ethiopia since it was taken from all regions and administrative towns. Despite its strength, the findings of the current study have limitations. Due to the use of secondary data, important variables like cultural beliefs in the postpartum period, Knowledge about postpartum danger signs were not available in the dataset, so these variables were not included in the analysis.

## Conclusion

The study also indicated that there was a statistically significant correlation between observations found in the same cluster. In addition to this, MOR for PNC utilization showed that there was variation between communities (clustering). Both individual and community-level factors were significant determinants of PNC utilization. From individual-level factors; the number of ANC visits, educational status of the husband and wealth index were significantly associated. From community-level factors, community level of education and community level of health service utilization was significantly associated with PNC service utilization. The educational status of a woman did not only affect the health of a woman herself, rather it goes to other women in that particular community. Based on the finding of this research, the following recommendations were forwarded: Design and implement policies that will strengthen women’s education at a different level of the population, improve the existing strategies of focused antenatal care for all pregnant women, increase and sustain utilization of all health services in all service delivery points, continuous and special attention should be given to the poorest population, emphasis should be given to the educational status of the husband, to strengthen health-seeking behaviors of the population, further qualitative research is needed to explore different cultural beliefs during the postpartum period which may prevent the women to seek PNC services, to conduct spatial analysis for giving appropriate area-specific intervention.

## Data Availability

The datasets used and/or analyzed during this study is available from the corresponding author on reasonable request.

## Abbreviations

CSA: Central Statistical Agency
EDHS: Ethiopia Demographic and Health Survey
PNC: Postnatal Care
WHO: World Health Organization
